# Great-tailed Grackles *(Quiscalus mexicanus)* as Biomonitors of Atmospheric Heavy Metal Pollution in Urban Areas of Monterrey, Mexico

**DOI:** 10.1007/s00128-021-03250-3

**Published:** 2021-05-08

**Authors:** Antonio Guzmán-Velasco, Javier I. Ramírez-Cruz, Gabriel Ruiz-Aymá, Iram P. Rodríguez-Sánchez, Lourdes Garza-Ocañas, Victor M. Treviño-Alvarado, José I. González-Rojas, Alina Olalla-Kerstupp

**Affiliations:** 1 Universidad Autónoma de Nuevo León, Facultad de Ciencias Biológicas, Laboratorio de Biología de la Conservación y Desarrollo Sustentable, C.P. 66455 San Nicolás de los Garza, Nuevo Leon México; 2 Universidad Autónoma de Nuevo León, Facultad de Ciencias Biológicas, Laboratorio de Fisiología Molecular y Estructural, C.P. 66455 San Nicolás de los Garza, Nuevo Leon México; 3 Universidad Autónoma de Nuevo León, Facultad de Medicina, Departamento de Farmacología y Toxicología, C.P. 66460 Monterrey, Nuevo Leon México; 4 Tecnologico de Monterrey, Escuela de Medicina, C.P. 64849 Monterrey, Nuevo Leon México

**Keywords:** Birds, Biomonitor, Feathers, Heavy metals, Air pollutants

## Abstract

Heavy metals exposure has been linked to severe health problems. In Mexico, the Monterrey metropolitan area (MMA) is considered one of the most polluted industrial regions. Because birds have been used successfully as environmental biomonitors, the levels of lead, cadmium, and arsenic in feathers of Great-tailed grackles (*Quiscalus* *mexicanus*) from two sites within and one site outside the MMA were determined. One hundred birds were captured, samples were analyzed by atomic absorption spectrometry. Ciudad Universitaria had the highest mean values of lead and cadmium, 11.91 ppm d.w. and 1.66 ppm d.w., respectively. This was at least, 10- and 8-times higher than the two other sample sites. We believe that using Great-tailed grackles as bioindicators in conjunction with air pollutants sampling from meteorological stations in Monterrey City could help in making decisions when applying environmental remediation measures as well as in the selection of places for housing, school and work among others.

Environmental pollution and chronic exposure to its components, including heavy metals, are considered a major public health problem, primarily because of their harmful effects on the ecosystems and the organisms that inhabit them (Valdez-Cerda et al. 2011; Parra-Ochoa 2014). The Monterrey Metropolitan Area (MMA) include 18 surrounding municipalities of the Nuevo Leon state and is located at Northeastern Mexico (25° 40′ N/100° 18′ W), almost 87% of the state population lives there (Blanco-Jiménez et al. 2015; INEGI 2020) and is considered an industrial area of great economic importance due to the variety of activities such as metallurgy, chemical, pharmaceutical, and textiles, to name a few (Valdez-Cerda et al. 2011). However, according to reports, the MMA is considered one of the most polluted industrial regions of Mexico with high levels of lead, cadmium, and arsenic in dust and streets (Green and Sanchez 2013; Badillo-Castañeda et al. 2015). Heavy metals, even at extremely low concentrations, are toxic and can promote the development of diseases (Sbarato et al. 1997; Valdez-Cerda et al. 2011; Badillo-Castañeda et al. 2015).

According to Anze et al. (2007), Hofer et al. (2010) and Parra-Ochoa (2014), humans and animals that share the urban environment, show similar responses at the biochemical and cellular level when they have been exposed to toxic agents in those environments. Due to the above, one strategy for evaluating environmental quality is the use of ecological indicators (Dale and Beyeler 2001). We chose to work specifically with the Great-tailed grackle (*Quiscalus* *mexicanus*) because this species meets the criteria of an ideal bioindicator: a bird, resident of the study area, not threatened, moderately abundant, relatively sociable with humans, easy to capture, with a body size that allows manipulation and effective collection of tissues (Koskimies 1989; Esselink et al. 1995; Padoa-Schioppa et al. 2006; Hofer et al. 2010; Parra-Ochoa 2014).

The MMA has 13 monitoring stations operated by the “Sistema Integral de Monitoreo Ambiental” [Comprehensive Environmental Monitoring System] (SIMA 2017) with the purpose of having up to-date information on the levels of pollutants, therefore, keeping the population informed about daily air quality. Although these air pollutants measurements by physicochemical methods are important, they do not allow conclusions to be drawn about the amount that can be absorbed by an organism nor the effects they can cause at the cellular or physiological level (Klumpp et al. 2004; Anze et al. 2007).

## Materials and Methods

Collections were carried out at three sites: two within the MMA and one outside the MMA (this last one presumably low in pollutants). MMA sites were the main university campus of the Universidad Autonoma de Nuevo Leon (Ciudad Universitaria, CU) in San Nicolas de los Garza (25° 43′ N/100° 18′ W) and the neighborhood Los Parques (LP), in Garcia (25° 47′ N/100° 27′ W). The one outside was in San Rafael (SR), a rural town belonging to Galeana, Nuevo León (25° 1′ N/100° 33′ W). Birds were captured using mist nets (Bub 1991; FAO 2007) between the months of May and October 2016. All birds were weighed and measured (the length of wing, tail, beak, and tarsus). Two rectrices were extracted from each specimen at the center of the tail because these usually persist longer in the bird’s body (Domínguez-Santaella 1998) and serve as a better parameter for tissue contaminant accumulation analysis (Nava-Díaz 2013). All birds were banded with a color plastic band to avoid analysis of recaptured birds and were released immediately into the wild.

The samples were analyzed in the Pharmacology and Toxicology Department of the Medical School of the Universidad Autonoma de Nuevo Leon. Determination of the concentration of heavy metals (lead, cadmium, and arsenic) was carried out by atomic absorption spectrometry (AAS).

The feathers were washed thoroughly with detergent and double distilled water to remove external contaminants; once dried they were cut into small pieces that were deposited in digestion tubes. Bidistilled water (3 mL) and 70% nitric acid (3 mL) were added to each digestion tube to dissolve the feather samples. Once dissolved, the content was placed in an oven and subjected to a pressure of 20 psi for 20 min. This process was performed three times to ensure complete digestion. Once the feathers were digested, each resulting solution was deposited in sterile plastic tubes.

For lead and cadmium determination the graphite furnace atomic absorption AAS technique was used, while the AAS-hydride heat generator was used to evaluate arsenic (Blanco Hernández 1998). Certified reference material (standard) was obtained from AccuStandard Inc. (New Haven, CT USA) and a calibration curve was made for each metal. The limit of quantitation (LOQ) and limit of detection (LOD) were 4 and 1.1 μg/mL (lead), 0.2 and 0.02 μg/mL (cadmium) and 2.5 and 1 μg/mL (arsenic).

To perform the reading of lead, 0.5 mL of the digested sample was deposited in 2 mL sample polystyrene cups for graphite furnace AAS and was directly analyzed; same was done for cadmium; however, an additional 0.5 mL dilution was needed (triton × 100 ammonium phosphate and nitric acid). For arsenic analysis, each sample was reacted with argon (Ar) gas and borohydride sodium (NaBH_4_), thus forming arsine (arsenic hydride, AsH_3_), to which heat was applied to break the molecule and power measure free arsenic.

To determine if there was a significant difference between the concentration of heavy metals and the localities, the Kruskal–Wallis test was applied. For the post-hoc analysis, the Jonckheere-Terpstra test was used; both tests with (*p* < 0.05) (IBM-SPSS Stadistics 20, 2011).

## Results and Discussion

**Fig. 1:**
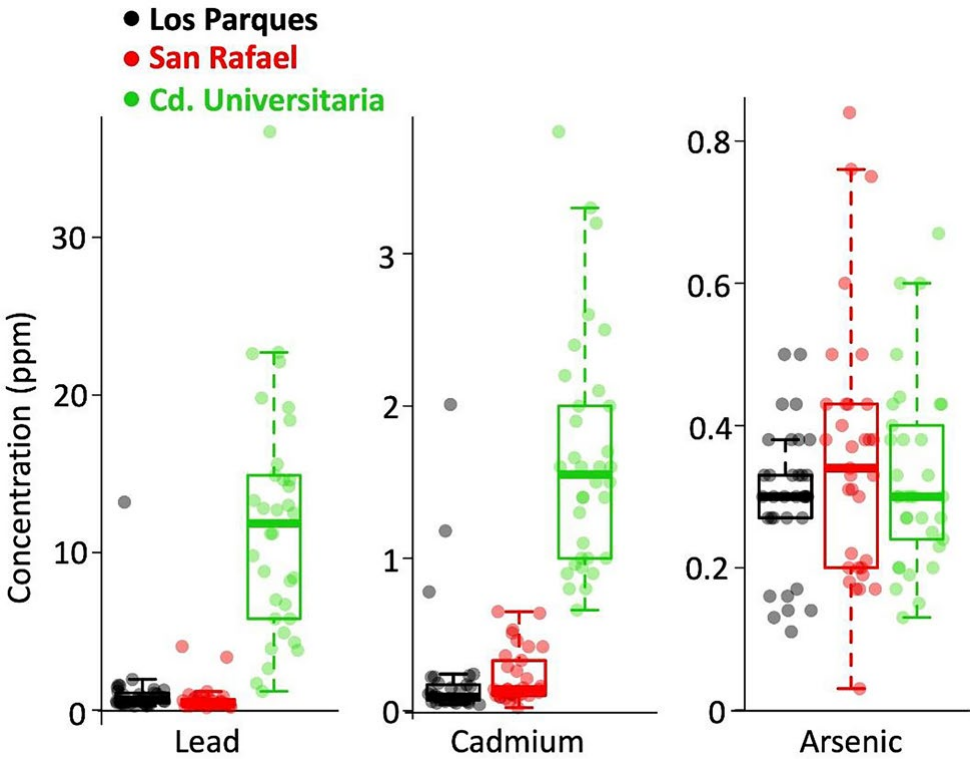
Comparison of the metal concentrations among collection sites. The vertical scale for each metal, in ppm, is indicated beside the dotted line (maximum ranged detected)

A total of 100 birds were captured; 34 were collected in CU (21 ♀♀/13 ♂♂), 33 in SR (19 ♀♀/14 ♂♂) and 33 in LP (25 ♀♀/8 ♂♂). The Kruskal–Wallis test indicated that, in the case of lead (χ^2^ = 65.84, *p* ≤ 0.05) and cadmium (χ^2^ = 64.40, *p* ≤ 0.05), there was a significant difference in concentrations between the sampled localities. On the other hand, there was no significant difference between sites for arsenic (χ^2^ = 2.06, *p* ≥ 0.05). The post-hoc comparison also indicated a significant difference between the locations for lead and cadmium [F(_2,3_) = 0.00, *p* < 0.05]; arsenic showed no significant difference [F(_2,3_) = 0.48, *p* > 0.05]. Concentrations for each heavy metal are shown in Table 1 and Fig. 1.

**Table 1:** Heavy metals concentration found in feathers of Great-tailed grackles by site and gender

Sites	Lead (ppm)		Cadmium (ppm)		Arsenic (ppm)	
	Mean ± SD (range)		Mean ± SD (range)		Mean ± SD (range)	
	♂♂	♀♀	♂♂	♀♀	♂♂	♀♀
CU	11.31 ± 9.76 (1.19–36.7)	12.29 ± 5.97 (1.7–22.7)	1.33 ± 0.52 (0.66–2.5)	1.26 ± 0.83 (0.8–3.8)	0.41 ± 0.12 (0.27–0.67)	0.28 ± 0.11 (0.13–0.6)
LP	0.74 ± 0.31 (0.26–1.19)	1.29 ± 2.53 (0.27–13.2)	0.31 ± 0.35 (0.04–1.18)	(0.05–2.01)	0.16 ± 0.05 (0.27–0.5)	0.34 ± 0.07 (0.27–0.5)
SR	0.75 ± 0.98 (0.19–4.04)	0.58 ± 0.71 (0.08–3.36)	0.27 ± 0.2 (0.09–0.65)	0.19 ± 0.14 (0.2–0.84)	0.41 ± 0.18 (0.2–0.84)	0.32 ± 0.017 (0.03–0.75)

*CU* Ciudad Universitaria, *LP* Los Parques, *SR* San Rafael

The use of internal tissue (lung, liver, kidney, muscle, etc.) has been an effective and accurate determination of heavy metal concentrations in birds (López et al. 2005). However, this method involves euthanizing the animals, which is why non-invasive techniques, such as blood and feather sampling, are more popular (Matz and Rocque 2007; Stout et al. 2010; Frantz et al. 2012). Feathers have been proven to be an excellent biomarker tissue of environmental contaminants (Markowski et al. 2013; Parra-Ochoa 2014). Levels of heavy metals in feathers reflect the circulating levels of these in blood during feathering (Burger et al. 2001; Johnston and Janiga 1995). However, one drawback is external contamination by atmospheric deposition and/or secretion by the uropygial gland in birds (Martínez-López et al. 2002; Dauwe et al. 2003). Furthermore, the concentration of contaminants may vary depending on the species, size and type of feather selected and if it has been newly molted (Dauwe et al. 2003). This can create an error in the concentration readings; by using a single of the same age (adult vs juvenile) as well as the same type of feather, and previous washing of the feather, we assume that the determined concentration of heavy metals corresponds to the contaminant absorbed by the feather.

The three heavy metals evaluated were detected in all feathers, which classifies them as ubiquitous pollutants (Bannon et al. 2011) and highlights their availability to birds on both urban and rural sites (Nava-Díaz 2013). Defining the limit at which the concentration of a contaminant detected can be considered toxic is difficult in birds as there are different species with significant variations (MMA 2006).

Various authors seem to agree with a normal range of 0.2 ppm to 0.6 ppm as a non-toxic concentration limit for lead in bird tissues (Getz et al. 1977; Grue and O’shea 1984; Burger et al. 2001; MMA 2006; Scheifler et al. 2006; Adout et al. 2007; Pan et al. 2008; Martorell 2009; Hofer et al. 2010; Frantz et al. 2012; Nava-Díaz 2013). As for cadmium, a range between 2 and 8 ppm has been mentioned as normal (EC 2001; Binkowski et al. 2013). In the case of arsenic, available literature mentions an upper limit in bird internal tissues of 1.67 ppm; above 10 ppm is considered toxic (Pan et al. 2008; Sánchez-Virosta 2015).

In our study, cadmium concentrations (1.66 ppm and 0.22 ppm for urban sites and 0.22 ppm in rural site) did not exceed the tolerance limit. Failure to register levels above the tolerance limit may be a good indication that for cadmium, the environmental situation of the collection sites is of good quality (Pan et al. 2008). As for lead, the two urban sites had a mean concentration of 11.91 ppm and 1.16 ppm, both above the tolerance limit of 0.2–0.6 ppm, whereas, in the rural area, a concentration of 0.66 ppm was determined, just on the edge of the upper limit. Our results correlate with the findings of Nava-Díaz (2013), Hofer et al. (2010), and Scheifler et al. (2006), who reported a higher concentration of heavy metals in urban sites (at least for cadmium and lead). Regarding arsenic, although we expected to find the same tendency of a lower concentration in the rural area, our results were slightly higher (0.36 ppm) in the rural than in the urban sites (0.30 ppm and 0.33 ppm). This increase may be due to fertilizers and pesticides that are used in crops of this site as arsenic is a component of these agrochemicals (Ferrer 2003; ATSDR 2007). Even so, the concentration of this metal in the three sites was below the 1.67 ppm tolerance upper limit, which makes us think that there is also no environmental emergency concerning arsenic.

The MMA is surrounded by mountains, which hinders air dispersion, and taking into account that the winds tend to go from east to west (INECC 2007), this means that air pollution tends to disseminate towards the municipality of Garcia, so it was expected that the feathers of the grackles collected in Los Parques, presented a higher concentration of the three heavy metals; however and surprisingly, these were lower than those of Ciudad Universitaria which is located in the northeast portion of the MMA.

As we have highlighted, within the MMA, Ciudad Universitaria obtained the highest levels of 2 of the 3 pollutants (lead and cadmium). This may be due to the presence nearby of different industries dedicated to the manufacture, transformation and commercialization of pigments, dyes, chemical products and steel, among others. Another possible contaminating factor within Ciudad Universitaria is vehicle traffic. The university is located between two major avenues which have a high concentration of motor vehicles throughout the day, especially during rush hour.

The prevalence of heavy metal pollution and mobility of lead and cadmium was investigated in street dust samples from the MMA in 2011. Eleven samples were taken within a radius of 5 km around Ciudad Universitaria. All samples contained both heavy methals and were mostly attributed to vehicle emissions and residual forms of different speciation. The majority of lead was associated with the residual fraction followed by the carbonate fraction; the majority of cadmium was associated with the residual fraction. The results indicated that mobility was higher in lead compared with cadmium, posing a potential risk to the environment (Valdez-Cerda et al. 2011).

In the municipality of Garcia, there are also many industries of different kinds, however, we believe that a factor that may have influenced in recording lower heavy metals concentration in Los Parques vs Ciudad Universitaria is bird migration. Birds were caught in Los Parques during October. Although the grackle is a resident bird, there are some northern populations (AOU 1998; Howell and Webb 1995) that partially migrate during this time. During these small migrations, birds tend to settle in public parks, facilitating their capture; however, is possible that we captured individuals that might not have been residents for enough time to reflect the actual concentration levels in their feathers.

There was no significant difference in the lead and cadmium concentrations in the feathers of females and males. This could be due to the time of formation of feathers, which takes an average of 20 days in both females and males (Cassan 2006). As for arsenic, the feathers of females had a higher concentration than males. Also, the concentration of this pollutant was higher in females of rural areas. Some captures were made in the breeding season (egg formation), this probably increases the amount of water and food ingested by the females and could explain why they presented higher concentrations. Arsenic is mainly absorbed through ingestion (Ferrer 2003; ATSDR 2007); therefore, we can infer that the increase of arsenic in their system is due to the consumption of irrigation water and/or the consumption of fertilized seeds and insects fumigated with arsenic-based components.

Despite the decrease in atmospheric lead concentrations in urban areas of most industrialized countries (Scheifler et al. 2006), this heavy metal is one of those found at higher levels with higher frequency in bird tissues. Like other metallic trace elements, lead is non-degradable and due to past high emissions, and we must say, to current emissions from activities such as metallurgy, dyes, and battery production, among others, urban soils will remain contaminated for a long time. This reflects the importance of this metal in urban environment pollution (Nam and Lee 2006a, b; Swaileh and Sansur 2006; Naccari et al. 2009; Parra-Ochoa 2014).

Our results demonstrate that Great-tailed grackles in the MMA had important levels of lead in their tissues (feathers). If birds have a high level of lead in their system, it is expected that other organisms, including humans, may show a similar effect. This, in turn, may trigger health disorders due to a longer exposure time.

Concentrations of heavy metals in bird feathers (specifically, in the Great-tailed grackle) have never been assessed before in the MMA. Results presented here are part of a pilot plan for continuous monitoring within the MMA.

We believe this type of work and complemented by physicochemical studies of environmental pollutants and physiological response to heavy metal exposure analysis, both in birds and humans, could help in making decisions when applying environmental remediation measures as well as in the selection of places for housing, school, and work, among others.
